# The Establishment of a Hyperactive Structure Allows the Tumour Suppressor Protein p53 to Function through P-TEFb during Limited CDK9 Kinase Inhibition

**DOI:** 10.1371/journal.pone.0146648

**Published:** 2016-01-08

**Authors:** Thomas K. Albert, Claudia Antrecht, Elisabeth Kremmer, Michael Meisterernst

**Affiliations:** 1 Institute of Molecular Tumor Biology (IMTB), Faculty of Medicine, University Muenster, 48149, Muenster, Germany; 2 Institute of Molecular Immunology (IMI), Helmholtz Research Center for Environment and Health, 81377, Munich, Germany; Baylor College of Medicine, UNITED STATES

## Abstract

CDK9 is the catalytic subunit of positive elongation factor b (P-TEFb) that controls the transition of RNA polymerase II (RNAPII) into elongation. CDK9 inhibitors block mRNA synthesis and trigger activation of the stress-sensitive p53 protein. This in turn induces transcription of CDKN1A (*p21*) and other cell cycle control genes. It is presently unclear if and how p53 circumvents a general P-TEFb-requirement when it activates its target genes. Our investigations using a panel of specific inhibitors reason for a critical role of CDK9 also in the case of direct inhibition of the kinase. At the prototypic *p21* gene, the activator p53 initially accumulates at the pre-bound upstream enhancer followed—with significant delay—by *de novo* binding to a secondary enhancer site within the first intron of *p21*. This is accompanied by recruitment of the RNAPII initiation machinery to both elements. ChIP and functional analyses reason for a prominent role of CDK9 itself and elongation factor complexes PAF1c and SEC involved in pause and elongation control. It appears that the strong activation potential of p53 facilitates gene activation in the situation of global repression of RNAPII transcription. The data further underline the fundamental importance of CDK9 for class II gene transcription.

## Introduction

The tumor suppressor protein p53 becomes activated and stabilized upon cellular stress and various types of genotoxic insult. The p53 response is tightly controlled and fine-tuned at multiple levels, for example by stimulus-specific posttranslational modifications of p53 that affect its stability and/or its interactions with other proteins and DNA. After nuclear translocation, p53 acts primarily as transcriptional activator that binds as tetramer to *cis*-regulatory regions of genes involved in cell cycle arrest, senescence, DNA repair or apoptosis [1]. The initial characterization of a p53-specific response element (RE) dates back to the early 1990s [2,3]. The p53 RE consensus comprises two copies of the sequence 5'-Pu-Pu-Pu-C-A/T-T/A-G-Py-Py-Py-3' (with Pu and Py indicating purines and pyrimidines, respectively), which are separated by a spacer of varying length. One of the first identified p53 REs was found 2.4 kb upstream of the transcription start site (TSS) of the cell cycle control gene *p21* and functions as enhancer that mediates transcriptional activation of *p21* [4]. Since then, the number of validated p53 target genes has increased significantly, and, based on recent genome-wide gene expression and mapping studies, current estimates on the scale of the p53 transcriptional program range from several hundred to thousand direct p53 target genes [5–9].

A recent study uncovered another regulatory layer of *p21* gene activation: a p53-induced long non-coding (lnc) enhancer RNA termed LED was found to associate with a hitherto uncharacterized p53 RE in the first intron of *p21* [10]. LED binding contributes to *p21* activation, and the intronic element was shown to bear enhancing potential in reporter assays. Thus, *p21* gene expression appears to be regulated by at least two separate enhancers.

Transactivation by p53 involves the recruitment of general transcription factors (GTFs) of the RNAPII initiation machinery, e.g. TFIIA, TFIID or TFIIH, to the core promoter region of target genes [11]. Beyond initiation, p53 interacts with several factors that are involved in subsequent phases of the RNAPII transcription cycle, such as promoter escape or transcription elongation. For example, in an *in vitro* chromatin transcription system p53 was shown to cooperate in a dose-dependent manner with purified human PAF1 elongation factor complex (PAF1c) [12]. Moreover, physical interactions of p53 with cyclin-dependent kinase CDK9 have been reported [13,14]. CDK9 is the catalytic subunit of P-TEFb, a key mediator of RNAPII pause release that is a major rate-liming step of mRNA synthesis in metazoans [15]. The major role of P-TEFb is phosphorylating DSIF (DRB sensitivity-inducing factor) and NELF (negative elongation factor)—two factors that stabilize paused polymerases *in vivo*—to relieve their negative impact on RNAPII progression into more downstream regions. Inhibition of CDK9's catalytic activity by small molecules such as flavopiridol (FP) or 5,6-dichloro-1-beta-D-ribofuranosyl-benzimidazole (DRB) blocks mRNA synthesis in living cells, which in turn triggers p53 activation [16,17]. A subset of p53 target genes, including *p21*, escape from transcriptional repression [18].

Here we have addressed the unresolved issue whether p53 bypasses or functions through P-TEFb like other activators. Our data show that the latter is the case. Transactivation by p53 can only prevail over generic repression when P-TEFb blockage is incomplete. The tumor suppressor uses its strong transactivation potential to hyperactivate the prototypic *p21* gene. This process involves recruitment of transcription initiation and elongation factors including Mediator, the PAF1 and the Super Elongation Complex (SEC). Loss of these factors attenuates inducibility of *p21* upon transcription stress. Beyond it, we suggest that cumulative loading of p53 onto the two enhancers of *p21* confers particular responsiveness to this gene.

## Material and Methods

### Cells

MCF7, A549, HeLa and 293T cells were obtained from DSMZ (Braunschweig, Germany) or ATCC (Manassas, VA, USA) and cultivated in DMEM medium supplemented with L-glutamine (2 mM), penicillin-streptomycin (100 units/ml-100 μg/ml; all from Life Technologies, Darmstadt, Germany) and 10% FBS (FBS Gold; GE Healthcare).

### Short interfering (si) RNA transfection

MCF7 cells were transfected using Lipofectamine 2000 (Life Technologies) at approximately 25% confluency with siRNAs pools for ENL (M-016352-01), ELL (L-008176-00), CDK9 (L-003243-00), PAF1 (M-020349-01), MED26 (M-011948-02) and non-targeting control (D-001810-10; all from Dharmacon/GE Healthcare) or a single siRNA for CDK12 (sc-44343; Santa Cruz Biotechnology, Dallas, TX, USA) at a final concentration of 40 nM.

### Antibodies and inhibitors

Antibody reagents used in this study included antibodies from Santa Cruz Biotechnology: CDK8 (sc-1521), CDK9 (sc-484), CDK12 (sc-81834), cyclin T1 (sc-10750), ELL (sc-28702), MED26 (sc-48766 and sc-166614), p21 (sc-397), p53 (sc-126), p53 phospho-serine 392 (sc-56173), RNAPII (sc-899 and sc-55492), TFIIB (sc-225), alpha-Tubulin (sc-8035); from Bethyl Laboratories (Montgomery, TX, USA): AFF4 (A302-539A), BRD4 (A301-985A), CTR9 (A301-395A), ENL (A302-267A), LEO1 (A300-175A), RTF1 (A300-178A); from Cell Signaling Technology (Danvers, MA, USA): p53 phospho-serine 15 (#9286), p53 acetyl-lysine 382 (#2525); from Merck Millipore (Darmstadt, Germany): gamma-H2AX phospho-serine 139 (05–636); and from Abcam (Cambridge, UK): HEXIM1 (ab25388), rabbit IgG (ab46540). Rat monoclonal antibodies directed against CTD Ser2P (3E10) and Ser5P (3E8) were kind gifts from D. Eick (Helmholtz Center Munich). The rat monoclonal antibody against SPT5 (6F1) was generated using a synthetic peptide with the amino acid sequence PLQDGSRTPHYGSQTPLH derived from the human SPT5 C-terminal region. Flavopiridol (F3055), DRB (D1916) and Nutlin-3 (N6287) were obtained from Sigma-Aldrich (St. Louis, MO, USA), and 067 was synthesized and purified as described previously [19]. Inhibitor stocks were prepared in DMSO and stored in the dark at -20°C until use.

### Reverse transcription-quantitative PCR (RT-qPCR)

Reverse transcription of total RNA prepared by Trizol (Life Technologies) was carried out using the PrimeScript RT kit (Clontech Laboratories, Mountain View, CA, USA), and cDNAs were analyzed by quantitative real-time PCR (qPCR) using Power SYBR Green PCR Master mix on a Step One Plus PCR system (Life Technologies). Fold changes of transcripts were calculated by the delta-delta C_T_ method. Primers for detection of spliced mRNA or unspliced pre-mRNA were designed with the program Primer3 (http://primer3.wi.mit.edu/).

### Chromatin immunoprecipitation (ChIP)

ChIP was performed as described previously [19]. ChIP and input DNAs were purified with the QIAquick PCR purification kit (Qiagen, Hilden, Germany). Serial dilutions of input DNAs were used as standards, and qPCR was carried out as above.

### Immunoblot (IB) analyses

Whole cell extracts were prepared with GENNT lysis buffer [5% glycerol, 5 mM EDTA, 0.2% Igepal CA-630, 150 mM NaCl, 50 mM Tris–HCl pH 8.0, 1x Complete protease inhibitor (Roche, Mannheim, Germany)]. After sample electrophoresis and transfer to PVDF membrane, the immunoblots were incubated with primary and secondary antibodies and developed using Western Lightning ECL (Perkin Elmer, Akron, OH, USA) and Hyperfilm ECL (GE Healthcare).

### Nuclear extracts and immunodepletion

Nuclear extracts (NEs) of MCF7 and 293T cells were essentially prepared as described previously [20]. NEs were dialyzed against buffer BC0 (20 mM Tris HCl pH 7.3, 20% glycerol, 0.2 mM EDTA, 1 mM PMSF and 5 mM DTT) and KCl was added to a final concentration of 100 mM (BC100). NEs were immunodepleted with Dynabeads Protein A or G (Life Technologies) as described previously [20].

### *In vitro* transcription

*In vitro* transcription reactions were performed on immobilized DNA templates as described [20,21]. Transcription reactions contained 150 μg NE and 5 pmol recombinant Gal4-VP16. After PIC assembly (60 minutes) and elongation (5 or 12 minutes) at 25°C, reactions were stopped by the addition of 2 volumes of 40 mM EDTA, beads were washed with buffer WB1 (25 mM HEPES pH 8.2, 5 mM EDTA, 1 mM DTT, 0.2 mM EDTA pH 8.0, 0.01% Igepal CA-630, 70 mM potassium glutamate, 10% glycerol), RNA purified and analyzed by autoradiography.

## Results

### The inhibition of CDK9 leads to initial repression and subsequent strong induction of the *p21* gene by p53

**Fig 1:**
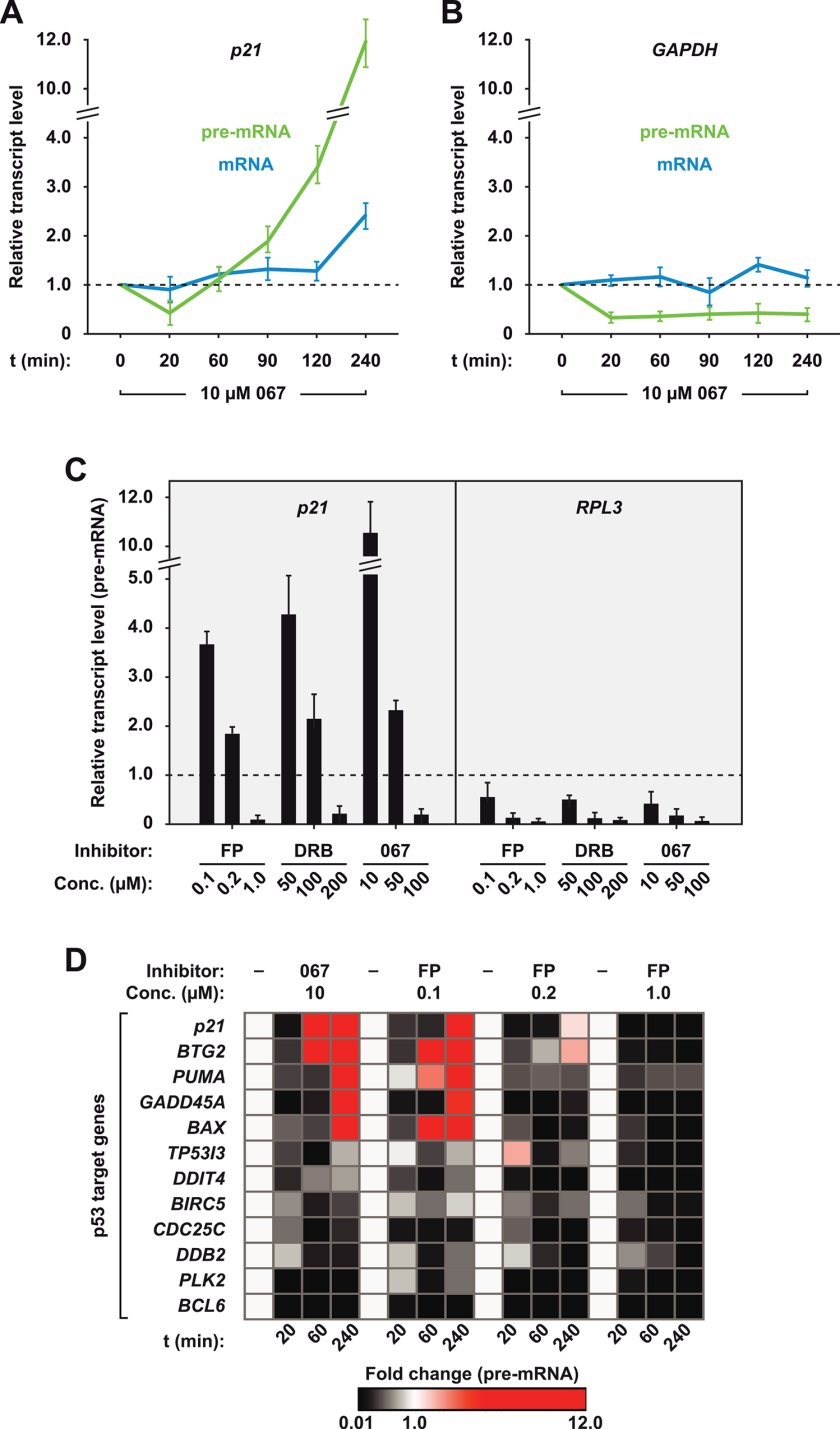
P53 target gene response to CDK9 inhibition. (A) Kinetic effects of CDK9 inhibitor treatment on mRNA synthesis of *p21*, and of *GAPDH* (B). Total RNA from 067-treated MCF7 cells was subjected to RT-qPCR analysis and normalized to untreated cells. Mean and standard error of mean (s.e.m.) values of biological triplicates are shown and presented as relative changes of transcript levels. (C) Dose-dependent effects of CDK9 inhibitors on pre-mRNA synthesis of *p21* and *RPL3*. (D) P53 target gene response to CDK9 inhibition. Total RNA from DMSO-treated (-) or inhibitor-treated MCF7 cells was analyzed as in (A), and relative changes of pre-mRNAs are presented as heat map using GENE-E (http://www.broadinstitute.org/cancer/software/GENE-E/).

The proposed global function of CDK9 in RNAPII transcription led us to hypothesize that inhibitors directed against the kinase CDK9—albeit leading to activation of p53—would also limit the transcriptional activation of p53 target genes. We further assumed that activation of p53 by ATM/ATR kinase family members follows CDK9 inhibition with a certain delay because the former sense the impaired pause release of RNAPII induced by inhibition of the latter [22]. To clarify this issue mRNA synthesis was kinetically dissected at p53 target genes following the addition of CDK9 inhibitors. Here we employed three different inhibitors, DRB, Flavopiridol (FP) and 067, with distinct affinity towards the ATP binding pocket of CDK9 (DRB < 067 <FP [19]; see also S2 Fig for activation of p53 by the inhibitors). Among them, the previously characterized intermediate-affinity compound 067 [19], a 2,4-amino pyrimidine derivative, proved especially instructive. MCF7 cells, which harbour functional wild-type p53 [23], were treated for 20 minutes to 4 hours with 10 μM 067 and both nascent unspliced precursor (pre) and mature spliced mRNA was analyzed by quantitative PCR (qPCR). Inhibitor treatment led to significant (2.5-fold) repression of nascent RNA synthesis of the p53 target gene p21 within 20 minutes (Fig 1A). The levels of pre-mRNA increased steadily thereafter, resulting in roughly recovery to initial levels after 1 hour and a 12-fold induction after 4 hours. At this time point steady-state levels of mature *p21* mRNA began to rise, too. In contrast, the time-course of repression of the housekeeping gene *GAPDH* showed rapid saturation within approximately 20 minutes and, unsurprisingly, no recovery in the subsequent hours (Fig 1B). As expected, the increase of *p21* mRNA was entirely due to transcriptional activation by p53: it was also observed in p53-positive A549 cells but not in HeLa and HCT116 p53-/- cells that lack functional p53 (S1 Fig). Together, these data indicate that p53 target genes are subject to repression following attenuation of CDK9 like non-target genes. The response of p53 target gene *p21* is biphasic in nature. In principle, the final activated state of *p21* after several hours could reflect a change in the transcription process (for example by switching from a CDK9-dependent to an independent mechanism) or simply reflect the superposition of both processes—i.e. incomplete repression and strong activation—which we addressed below.

### P53 transactivation functions through CDK9

We tested whether p53-dependent target gene activation overcomes higher doses of 067 and other CDK9 inhibitors. Titrations of 067, FP and DRB in MCF7 cells revealed a dose-dependent switch of the *p21* transcriptional response, which ranged from 5-fold repression at the highest dose to over 4-fold induction at the lowest dose of each inhibitor (Fig 1C). Dose-dependent effects of the three compounds were also observed on protein level (S2 Fig). In contrast to *p21*, mRNA synthesis of the p53 non-target gene *RPL3* was exclusively repressed in a dose-dependent manner by all three inhibitors (Fig 1C).

Other target genes in the p53 program behaved similar to *p21*. Both partial CDK9 inhibition (by 10 μM 067 or 0.1 μM FP) or full inhibition (by 1 μM FP) led to repression of all 12 interrogated genes after 20 minutes, and this repression persisted when CDK9 activity was fully blocked by 1 μM FP (Fig 1D). Yet, sub-saturating concentrations of FP (0.1 μM) or 067 (10 μM) allowed the recovery and subsequent induction of 4 other p53 target genes besides *p21*: *BTG2*, *PUMA*, *GADD45A* and *BAX*. Collectively, these data show that transcription of p53 target genes functions through CDK9. The outcome of their transcriptional response depends on the degree of CDK9 blockage: if the kinase is completely inactivated, repression prevails, if residual CDK9 activity is retained, activation can overturn the initial repression.

### Transcription stress enhances assembly of transcription initiation complexes at the *p21* promoter

**Fig 2:**
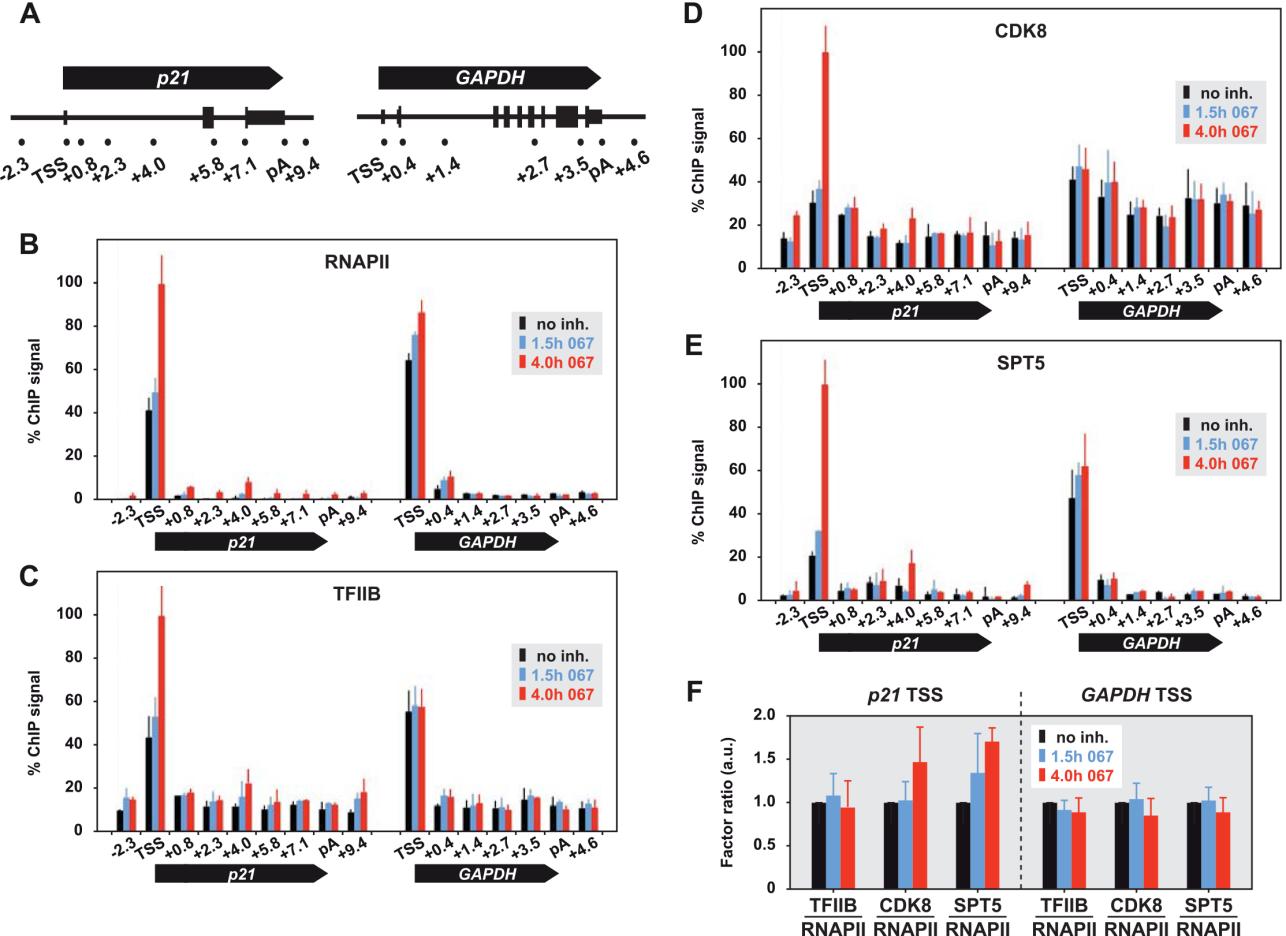
Recruitment of RNAPII, general initiation and pause release factors during transcription stress. (A) Gene structures of human *p21* and *GAPDH*. Locations and kilobase distance of ChIP-qPCR amplicons to the transcription start site (TSS) are shown underneath. pA, polyadenylation site. (B) ChIP profiles of RNAPII, (C) TFIIB, (D) CDK8, and (E) SPT5 across *p21* and *GAPDH* loci in DMSO-treated MCF7 cells (black bars; no inhibitor) or or cells treated with 10 μM 067 for 1.5 hours (blue bars) and 4 hours (red bars). The maximal signal within each ChIP was set to 100%. Mean values ± s.e.m. (2 biological replicates, 3 qPCR reactions per sample) are shown. (F) Normalized factor stoichiometries at the TSS of *p21* or *GAPDH*.

It has been shown that in non-stressed cells the *p21* gene is pre-loaded with basal levels of p53 at a distal enhancer and with poised RNAPII at the core promoter. Stress induces the accumulation of p53 at the enhancer and the conversion of stalled RNAPII into an elongation-proficient form [18,24]. We asked how transcription initiation is affected by compromised P-TEFb activity. RNAPII occupancy of the *p21* and *GAPDH* genes in control and 067-treated cells was analyzed by chromatin immunoprecipitation (ChIP). In the absence of inhibitor, RNAPII was strongly enriched at the transcription start site (TSS) of both genes, but barely detectable in intragenic regions (Fig 2B). Treatment with 067 for 1.5 hours led to the reinforcement of pausing as revealed by an approximately 1.2-fold increase of RNAPII ChIP signals at both promoters. After 4 hours, levels of promoter-bound RNAPII at *p21* exceeded those at *GAPDH* in terms of relative enrichment (2.5-fold versus 1.4-fold) as well as absolute quantities, indicating that the transcriptional induction of *p21* was accompanied by additional recruitment of RNAPII. The general transcription factor TFIIB, which is essential for the formation of the preinitiation complex (PIC) [25], showed a similar binding pattern to that of RNAPII (Fig 2C). The normalized ratio of the two factors remained unchanged in each condition, indicating that PIC stoichiometry was not affected by CDK9 inhibition (Fig 2F).

Another pivotal factor involved in eukaryotic gene transcription is the multi-subunit Mediator complex [26]. It has been shown previously that activated p53 employs the Mediator-associated kinase CDK8 as positive coregulator of p53 target genes [27]. We investigated how CDK8 recruitment relates to transcription stress-induced *p21* activation. CDK8 levels at the *p21* TSS increased above 3-fold upon inhibitor treatment and exceeded the relative increase of RNAPII at this location (Fig 2D and 2F).

Mediator couples transcription initiation and post-initiation steps through functional interaction with the SPT4/5 complex DSIF [28]. This pause factor associates with RNAPII in a transcription-dependent manner and turns into a positive elongation factor upon phosphorylation by CDK9. We asked if DSIF is bypassed upon P-TEFb blockage. ChIP of SPT5 with a newly developed, functionally proven antibody showed nearly perfect colocalization with RNAPII in each condition, indicating that the tight association of SPT5 with elongating polymerase was preserved in the presence of 067 (Fig 2E).

### *P21* induction requires elongation factor complexes SEC and PAF1c

**Fig 3:**
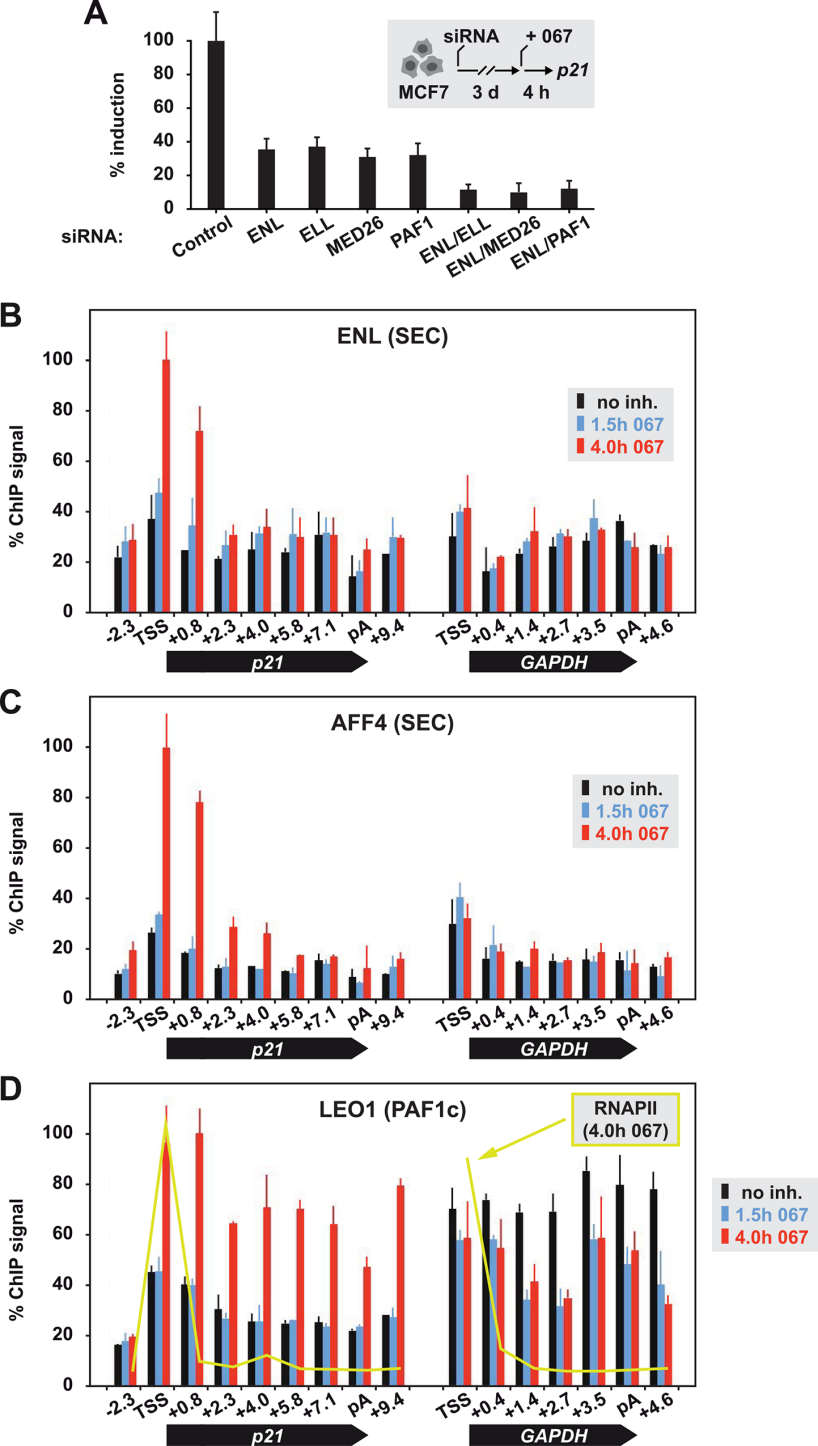
Involvement of SEC and PAF1c in *p21* activation. (A) MCF7 cells were transfected with the indicated siRNAs and after three days treated with 10 μM 067 for 4 hours. Effects on *p21* mRNA synthesis were analyzed by RT-qPCR. Induction levels of *p21* in control samples were set to 100 percent (mean + s.e.m. of biological triplicates). (B) ChIP profile of SEC subunit ENL, (C) AFF4, or (D) PAF1c subunit LEO1 across *p21* and *GAPDH* gene loci. In (D), the binding profile of RNAPII after 4 hours treatment with 067 is shown for comparison (lime line).

The potential bypass of CDK9 function by p53 predictably involves elongation factors. We focussed on the Super Elongation Complex (SEC), which had not been implicated in p53-dependent gene control until now, and the PAF1 complex (PAF1c). Individual components of SEC and PAF1c were depleted by short interfering (si) RNA-mediated knockdown. They included ENL and ELL, the PAF1 protein and the Mediator subunit MED26, which physically bridges SEC and RNAPII [29] (S3 Fig). Treatment of knockdown cells with 067 showed that SEC, MED26 and PAF1 were rate-limiting for full-level activation of *p21* in conditions of transcription stress (Fig 3A). This prompted us to investigate the distribution of these factors at the *p21* gene. SEC subunits ENL and AFF4 colocalized with each other and became enriched 3- to 4-fold in the TSS and proximal downstream region of *p21* in the late activation phase (Fig 3B and 3C). SEC occupancy of the *GAPDH* gene remained on basal levels. Different from it, the PAF1c component LEO1 correlated with the transcriptional response of both genes: it increased at *p21* and decreased at the repressed *GAPDH* gene upon treatment with 067 (Fig 3E). Relative to RNAPII, more LEO1 protein was retrieved from promoter-distal regions by ChIP, suggesting that PAF1c dissociates from the polymerase during elongation.

### Ser2P is controlled by CDK9 but uncoupled from transcription activation

**Fig 4:**
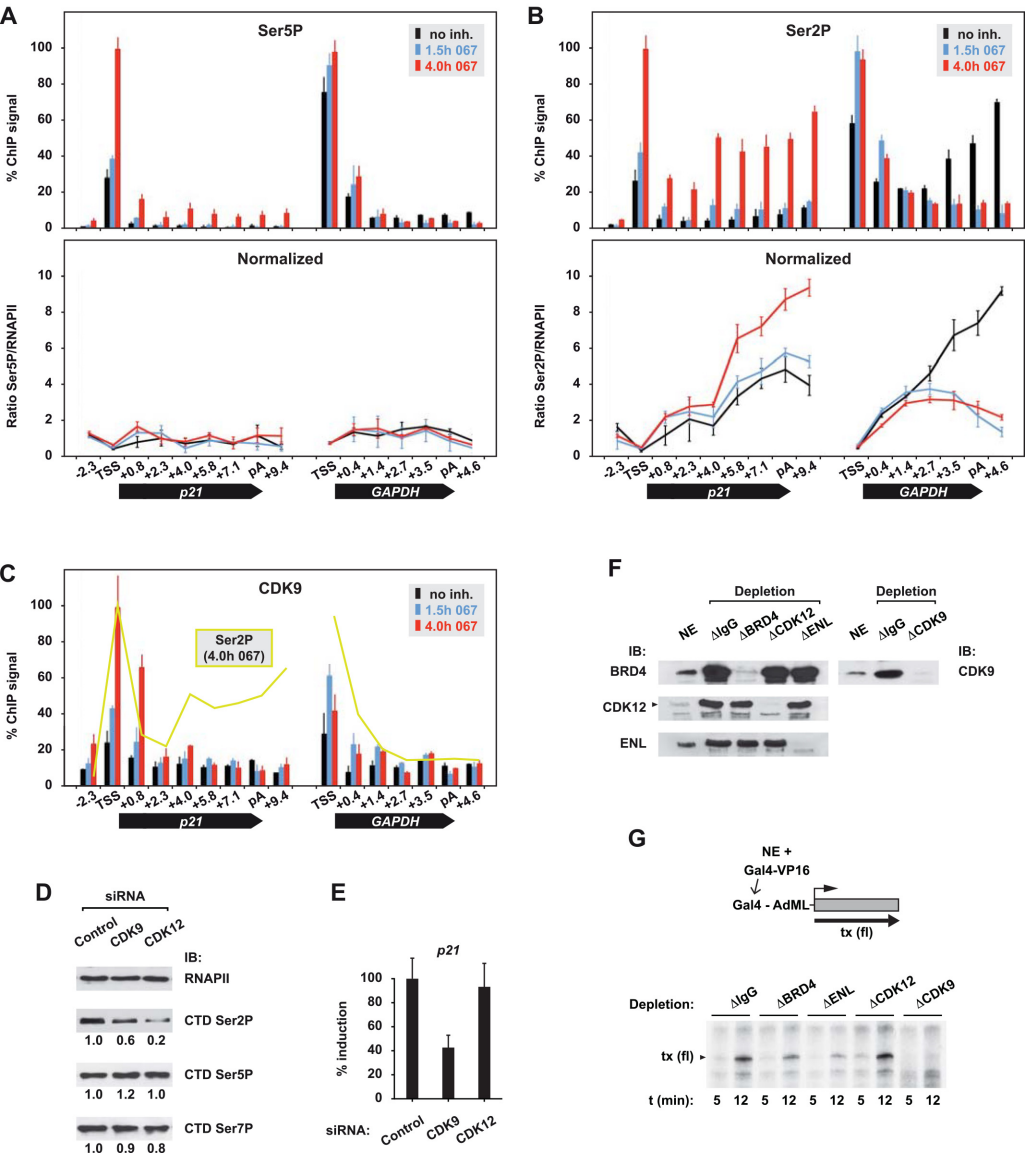
CDK9 controls Ser2P and is essential for activator-driven transcription. (A) ChIP profile of CTD Ser5P, and (B) Ser2P across the p21 and *GAPDH* loci in the absence or presence of 067. Lower graphs show relative levels of CTD modifications after normalizing to RNAPII. (C) ChIP profile of CDK9 (and, for comparison, of Ser2P). (D) Immunoblot (IB) analysis of RNAPII and bulk levels of Ser2P/5P/7P in control, CDK9 and CDK12 knockdown cells. Band intensities of CTD modifications were determined using ImageJ and normalized to RNAPII in the control sample. (E) Effect of CDK9 or CDK12 knockdown on *p21* inducibility was determined as in Fig 3A. (F) Nuclear extracts (NE) from 293T cells were immunodepleted (□) with non-specific IgG or the indicated antibodies and analyzed by immunoblotting (IB). (G) Depleted extracts were used in *in vitro* transcription reactions together with recombinant Gal4-VP16 activator and a responsive PCR template containing the Adenovirus major late promoter (AdML). Transcription elongation continued for 5 or 12 minutes, and radioactively labeled transcripts (tx) were visualized by autoradiography.

There is an ongoing debate whether CDK9 phosphorylates serine at position 2 (Ser2P) or position 5 (Ser5P) of the RNAPII carboxyterminal domain (CTD). We used 067 to study CDK9-dependent RNAPII phosphorylation *in vivo*. As shown in Fig 4A, Ser5P overlapped perfectly with RNAPII, both in the absence and presence of 067. In contrast, inhibitor treatment led to a 3-fold reduction of Ser2P in the 3'-portion of *GAPDH* and a 2-fold increase of Ser2P in the 3'-half of *p21* (Fig 4B). Thus, CDK9 does not target Ser5 but controls phosphorylation of Ser2 in promoter-distal regions. The reason for gene-specific effects on Ser2P at the *p21* and *GAPDH* genes is unclear at present. It might involve context-dependent differences in P-TEFb activity at these two genes. In support of this idea, CDK9 occupancy followed SEC recruitment at *p21* but not *GAPDH* (Fig 4C). Since SEC harbors the catalytically most active fraction of nuclear P-TEFb [30], high-level recruitment of SEC/CDK9 could result in hyperphosphorylation of Ser2 at the *p21* gene. Yet, the divergent levels of CDK9 and Ser2P in the 3'-half of p21 point to another kinase that performs promoter-distal Ser2P *in situ* (Fig 4C). One likely candidate is CDK12, another CTD Ser2 kinase that was suggested to function downstream of CDK9 [31]. Knockdown of CDK12 led to a specific reduction of Ser2P bulk levels that was three times stronger than the one following knockdown of CDK9 (Fig 4D). However, while CDK9 was required for full-level induction of *p21*, CDK12 was non-essential for the activation process (Fig 4D). Together, the data reason for functional uncoupling of Ser2P from gene activation. Further supporting evidence was obtained from *in vitro* transcription assays with immunodepleted nuclear extracts (Fig 4F). While removal of CDK9 abolished activator-driven transcription (and removal of CDK9-interacting factors ENL or BRD4 impaired it), depletion of CDK12 had little effect in this system (Fig 4G).

### *P21* activation results in *de novo* recruitment of p53 to an intron enhancer

**Fig 5:**
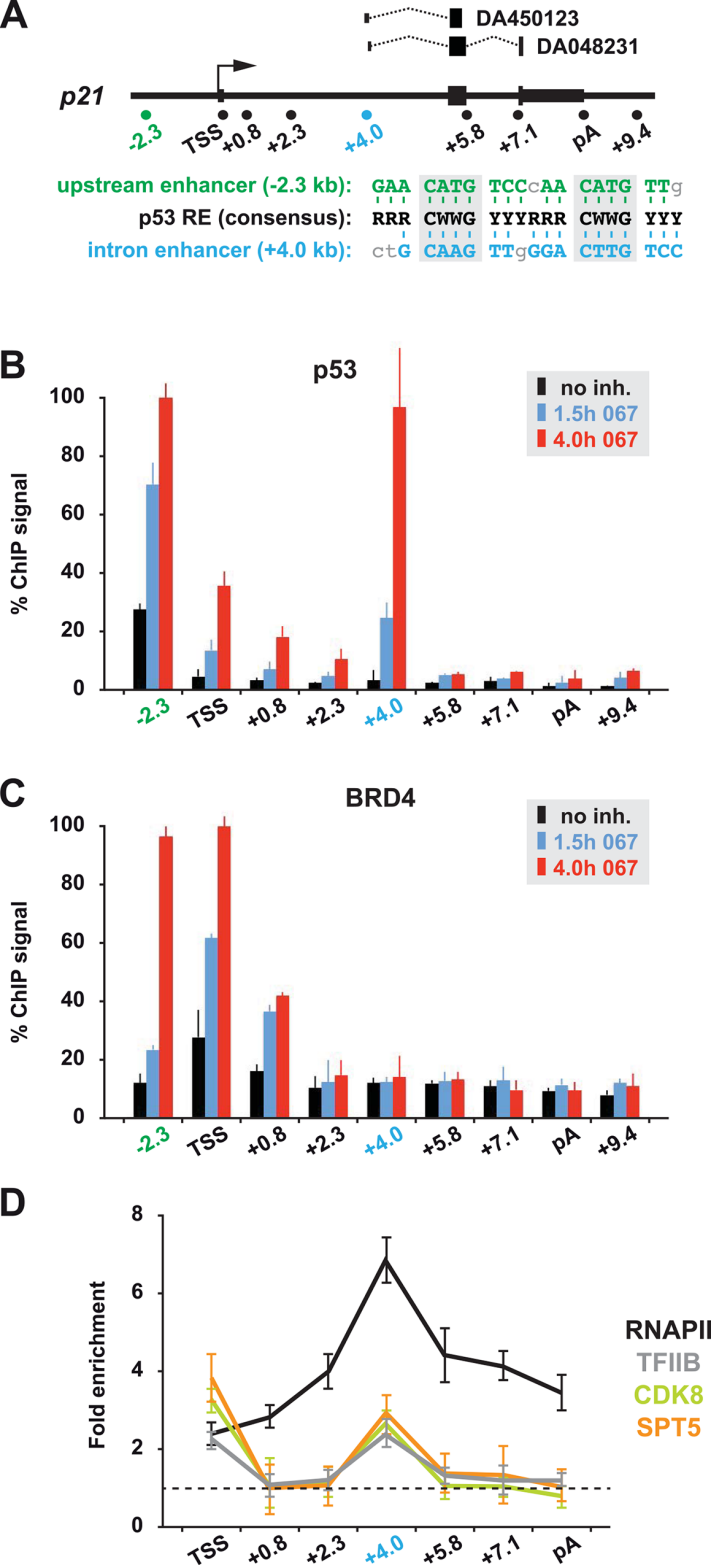
Association of p53 with a secondary enhancer site in the *p21* intron. (A) Map of the human *p21* gene. A sequence alignment of the consensus p53 response element (RE) with the upstream enhancer at position -2.3 kb and the intronic enhancer at position +4.0 kb is shown underneath, and the relative position and transcript structure of two spliced EST clones is indicated above. (B) ChIP profile of p53, and (C) of BRD4 across the *p21* gene in the absence or presence of 067. D. Local enrichment of the indicated factors within the transcription unit of *p21*. ChIP values of cells treated with 10 μM 067 for 4 hours were calculated as fold enrichment over DMSO-treated control samples (dashed line) for each position individually.

We reasoned that full-level induction of *p21* requires extensive loading of p53 onto *cis*-regulatory elements before the factor's transactivation capacity reaches a critical threshold that is sufficient to overcome P-TEFb blockage. Our ChIP experiments showed that prior to activation p53 was exclusively found at the upstream enhancer that harbors a high-affinity, near-consensus binding site for p53 (Fig 5A). Occupancy of this site increased incrementally after 1.5 and 4 hours of CDK9 inhibition (Fig 5B). In contrast to this pre-loaded "primary" enhancer, treatment with 067 was followed by *de novo* recruitment of p53 to additional, secondary binding sites, most notably to an intronic region that maps to position +4 kb downstream of the *p21* TSS and contains another near-consensus p53 motif (Fig 5A). After four hours of treatment, p53 occupancy of this site reached similar levels as found at the upstream enhancer (Fig 5B). Transcription stress also led to elevated levels of p53 at the TSS and in proximal downstream regions. Since those sequences are devoid of recognizable consensus motifs, p53 might get crosslinked to these regions indirectly, for example through DNA looping of the upstream enhancer and/or the intronic site.

For the upstream enhancer it has been previously shown that it is co-occupied in a mutually dependent manner by p53 and BRD4, a bromodomain protein, which interacts with p53 and positively regulates *p21* transcription [32]. We asked whether p53-BRD4 co-occupancy is also established at the intronic element. This was not the case: while ChIP of BRD4 revealed a positive correlation with p53 levels at the upstream enhancer, the bromodomain factor was barely enriched at the intronic element (Fig 5C). However, delayed recruitment of p53 to this secondary binding site correlated with local enrichment of the RNAPII initiation/early elongation machinery (Fig 5D). The existence of p21-encoding Expressed Sequence Tags whose 5'-ends map to this position indicates that the intron element can serve as alternative transcription start site for shortened, yet functional *p21* transcripts (Fig 5A).

## Discussion

Pharmacological inhibition of the P-TEFb kinase CDK9 by small-molecule drugs leads to transcriptional activation of select p53 target genes such as *p21*, despite suppressing mRNA synthesis on a global scale. Our data show dose-dependency of this p53-mediated activation process: it only occurs if P-TEFb blockage is incomplete, while saturating inhibitor doses prevent it. Activation is delayed and follows an initial repression that also applies to p53 target genes. These findings illustrate that, instead of bypassing P-TEFb [18], p53 functions through CDK9 like other activators.

Transcriptional induction of *p21* involves the P-TEFb-containing Super Elongation Complex as well as PAF1c. These elongation factors are recruited to *p21* chromatin and needed for full induction of the gene. Although there are previous reports of p53-SEC and p53-PAF1c interactions [12,33], our results link these elongation factors for the first time with the p53-mediated stress response.

There are other examples of sequence-specific activators that engage both SEC and PAF1c to increase transcription rates of target genes. Most instructive in this regard is the HIV-1 protein Tat, a viral transactivator that utilizes P-TEFb as host cofactor [34]. Similar to p53-dependent *p21* activation, SEC and PAF1c are required and rate-limiting for Tat-dependent HIV-1 transcription [35]. Tat can interact with the CTD and enhance its phosphorylation [36], and it assembles and recruits Tatcom1, a higher-order complex of SEC and PAF1c, onto the viral LTR [35]. Our own unpublished data indicate that, in analogy to Tat, p53 associates physically with SEC and PAF1c in nuclear extracts of stress-challenged cells.

SEC and PAF1c knockdown combinations elicited stronger attenuation of *p21* induction than individual knockdowns, suggesting crosstalk of these two complexes. In further support of this idea, it has been shown that physical contacts with PAF1c connect SEC with RNAPII on chromatin [37]. Moreover, PAF1c and P-TEFb are linked through a positive feedback loop: SPT5 phosphorylation by P-TEFb helps to recruit PAF1c to active genes [38,39], and PAF1c *vice versa* enhances P-TEFb recruitment [40].

We also clarified the role of CDK9 and CTD Ser2P phosphorylation in gene activation: the selective CDK9 inhibitor 067 triggered changes of Ser2P—but not of Ser5P—on elongating RNAPII *in vivo*. This finding is in clear contrast to claims of CDK9 acting primarily as Ser5 kinase, which were mainly based on *in vitro* experiments using various CTD peptides as substrates [41]. It is, however, in perfect agreement with a recent study showing that treatment of cells with other CDK9-specific inhibitors, i.e. DRB or KM05283, results in reduced Ser2P (but not Ser5P) levels of RNAPII in the 3'-region of *GAPDH* [42]. These observations point to a strong context-dependency of CTD phosphorylation, which apparently occurs in a promiscuous manner *in vitro*, but turns into a highly selective process once the CTD is presented within functional transcription complexes.

Although CDK9 controls promoter-distal Ser2P *in vivo*, it is unlikely that the enzyme performs this task itself, because its binding to chromatin is limited to promoter-proximal regions. Another Ser2 kinase, CDK12, which requires upstream CDK9 activity, has been identified in *Drosophila* and human cells [31]. While CDK12 is not required for *p21* induction, CDK9 is rate-limiting. This indicates functional uncoupling of Ser2 phosphorylation and the activation process, and further suggests that generating Ser2P is not the essential role of CDK9. The differential impact of the two kinases on transcriptional activation extends beyond p53. We show it using a heterologous, artificial activator that drives *in vitro* transcription in nuclear extracts. It is also reminiscent of the differential requirements of CDK9 and CDK12 during germline development in *C*. *elegans*: while loss of CDK9 blocked this process completely, CDK12 had little impact on it [43].

The activity of P-TEFb is regulated by reversible association with the 7SK small nuclear ribonucleoprotein particle (snRNP) that also contains HEXIM proteins [44]. It has been previously shown that treatment of cells with the CDK9 inhibitors DRB and FP releases P-TEFb from the inhibitory 7SK snRNP, resulting in (at least transiently) increased availability of more free, active P-TEFb [45]. Our own preliminary experiments indicate that treatment of cells with 067 weakens the association of CDK9 with the 7SK snRNP component HEXIM1, thus indirectly reasoning for a similar effect of 067. In line with such a scenario, treatment of MCF7 cells with 067 leads to elevated levels of CDK9 in the *p21* promoter region, which might contribute to activation of the gene (Fig 4C).

The analysis of the *p21* locus further showed that delayed induction of *p21* upon treatment with 067 coincides with *de novo* recruitment of p53 to a binding site in the first intron of the gene. Inspection of genome-wide maps revealed p53 association with this element in other stress situations, e.g. when cells were exposed to the chemotherapeutic drug etoposide, while p53 was absent during neurogenic differentiation of human embryonic stem cells, in which the factor was otherwise occupying the upstream enhancer [6,46,47]. This suggests cumulative charging of *p21* with p53 as a stress-related mechanism to increase the transcriptional output of the gene. Indeed, a recent study discovered that the intronic element is functionally involved in a feed-forward loop that reinforces *p21* expression: it acts as enhancer, whose activity is stimulated by binding of LED, a long noncoding (lnc) enhancer RNA that itself is positively regulated by p53 [10]. The authors demonstrated that knockdown of LED impaired *p21* activation in MCF7 cells and that p53 and LED can bind concomitantly to the intronic enhancer region [10]. However, it remains unknown whether LED is required for recruitment of p53 (or vice versa) to the intron enhancer.

Collectively, our results highlight the involvement of SEC and PAF1c in transcriptional hyperactivation by p53, and point to topological reorganization of the *p21* regulatory domain as mechanism to overcome generic repression upon P-TEFb blockage.

## Supporting Information

S1 Fig *p21* activation in p53-positive versus p53-negative cancer cell lines. (EPS)

S2 Fig p53 and *p21* activation by CDK9 inhibitors. (EPS)

S3 Fig Knockdown of *p21* regulatory factors by siRNA. (EPS)
